# The effectiveness and safety of moxibustion for dry eye: Protocol for a systematic review

**DOI:** 10.1097/MD.0000000000015178

**Published:** 2019-04-12

**Authors:** Yingxin Zi, Meiqi Ji, Yu Deng, Yali Qin, Rui Wang, Huan Meng, Ming Jin

**Affiliations:** a Beijing University of Chinese Medicine; b Department of Ophthalmology, China-Japan Friendship Hospital, Beijing, China.

**Keywords:** dry eye, moxibustion, protocol, systematic review

## Abstract

**Background:**

Dry eye (DE) is a common type of ocular surface disease that affects many people. Moxibustion has been widely used in China to treat ocular surface diseases, especially DE. Many clinical trials have demonstrated that moxibustion can increase the tear secretion quantity and improve tear film stability. The purpose of this review is to provide an objective and normative systematic review to evaluate the effectiveness and safety of moxibustion treatment in patients with DE.

**Methods:**

The systematic review will include all of the randomized controlled trials on the effectiveness and safety of moxibustion for DE. Nine medical databases, namely PubMed, EMBASE, the Cochrane Library, Google Scholar, Web of Science, China National Knowledge Infrastructure (CNKI), China Science and Technology Journal database (VIP), Wanfang Database, and CBM, will be searched from the date of the database inception to February 28, 2019. We will also search registers of clinical trials, potential gray literature, and conference abstracts. There are no restrictions on language and publication status. Two reviewers will independently select studies, and extract and manage data. The reporting quality and risk of bias will be assessed by other 2 review authors. The primary outcomes will include ocular surface disease index (OSDI) scores, Schirmer test (SIT) results, tear meniscus height (TMH), and tear break-up time (TBUT) values. Quality of life, the main symptom scores before and after treatment, meibomian gland (MG) morphology, total treatment efficacy, and safety will be evaluated as the secondary outcomes. We will use RevMan V.5.3 software to synthesize and analyze data.

**Results:**

This systematic review will provide a comprehensive review of current evidence of moxibustion for DE from the following aspects: the OSDI, SIT, TMH, BUT, quality of life, the main symptom scores, MG morphology, total treatment efficacy, and safety.

**Conclusion:**

The conclusion of our systematic review will provide evidence to determine whether moxibustion is an effective and safe intervention for patients with DE.

**Ethics and dissemination:**

Since patients will not be involved in this study, it is not necessary to obtain ethical approval. The protocol and results will be published in a peer-reviewed journal.

**PROSPERO registration number:**

PROSPERO CRD42018097399.

## 1 Introduction

Dry eye (DE) is recognized as a multifactorial disease in which loss of dynamic balance of the tear film is the core pathophysiology concept.^[1]^ Inflammation plays a key role in the pathogenesis.^[2]^ DE has become the most common ophthalmic disease apart from ametropia in clinical ophthalmology.^[3]^ The incidence of DE in the world is 5.5% to 33.7%, and the incidence in China is 21% to 30%. DE patients in China account for more than 30% of ophthalmology outpatients.^[4]^ It has a complex pathophysiology and is multifactorial in nature.^[5]^ The common symptoms of DE include dryness, foreign body sensation, pain, redness, burning, and eye fatigue.^[6,7]^ As a chronic ophthalmic disease, DE, which in serious cases might even lead to blindness, not only affects the quality of life of the patient but also poses adverse impacts on social productivity and work efficiency, and influences the development of society and the economy.^[8]^ The prevalence and morbidity of DE have been rising in recent years because of the widespread use of video terminals, which pose greater challenges to people's vision than other lifestyle choices. With changes in the social environment and people's lifestyles, the prevalence of DE is increasing.^[9]^ Tear film is composed of 3 layers: a mucin layer lies in the innermost, a central aqueous layer is the thickest, and an outermost lipid layer covers ocular surface.^[10]^ DE is classified into aqueous tear deficiency DE, evaporative DE, mucin-deficient DE, tear dynamic abnormal DE, and hybrid DE.^[11]^

The treatment for DE encompasses both medical therapy and operative treatment, including avoidance of exacerbating factors, eyelid hygiene, tear supplementation, tear retention, tear stimulation, anti-inflammatory agents, lacrimal punctal or passage occlusion, and surgical therapies.^[12]^

At the same time, complementary and alternative medicine therapeutics for treating DE is increasing in popularity.^[13,14]^ A previous systematic review showed that acupuncture therapy is more effective than artificial tears (ATs) for DE syndrome.^[15]^ As an important part of Traditional Chinese Medicine (TCM) and a natural therapy, moxibustion has a wide range of indications and plays a positive effect on some chronic and severe diseases (eg, chronic kidney disease and allograft nephropathy).^[16–18]^ The start-up mechanism of moxibustion warming and dredging function is a continuous and complex process,^[19,20]^ which works by stimulating acupuncture points with igniting the heat of moxibustion.^[16]^ In the DEWS II report, a new chapter on the role of alternative treatment (Chinese medicine, acupuncture, and moxibustion) in DE disease is presented.^[21]^ Jiang et al^[22]^ demonstrated that the effect mechanism of warming-dredging in moxibustion lies in anti-inflammation, which could related to transient receptor potential vanilloid (TRPV). The population of patients with DE is tending to become younger, and especially includes those with allergic diseases.^[23]^ Heat released by moxibustion can induce adenosine triphosphate release from mast cells (MCs), and the initiating response of acupoints to moxibustion may lie in purinergic signals originating from MCs.^[24]^ However, to our knowledge, there is a lack of critically designed systematic review to evaluate the effectiveness of moxibustion for DE. In this study protocol, we will present the protocol and assess all of the clinical evidence on the effectiveness and safety of moxibustion for DE patients.

## 2 Methods

The systematic review protocol has been registered on PROSPERO (https://www.crd.york.ac.uk/PROSPERO/#recordDetails, registration number: CRD42018097399).^[25]^ Our protocol will follow the Cochrane Handbook for Systematic Reviews of Interventions and the Preferred Reporting Items for Systematic Reviews and Meta-Analysis Protocol (PRISMA-P) statement guidelines.^[26,27]^

### 2.1 Inclusion criteria for study selection

#### 2.1.1 Types of studies

We will consider only clinical randomized controlled trials (RCTs) of moxibustion therapy for DE. We will exclude non-RCTs, quasi-RCTs, uncontrolled trials, reviews, case-controlled studies, case reports, animal trials, and laboratory studies.

#### 2.1.2 Types of patients

Patients diagnosed as having DE will be included. The definitions of DE or dry eye syndrome (DES) or keratoconjunctivitis sicca will be included. There will also be no restrictions based on other conditions, such as age, sex, race, educational or economic status, disease duration, and disease severity.

#### 2.1.3 Types of interventions

Studies that evaluated any form of moxibustion (eg, direct moxibustion, indirect moxibustion, heat-sensitive moxibustion, moxa burner moxibustion, warm needling, crude drug moxibustion, or natural moxibustion) will be included.^[16]^ We will exclude trials that moxibustion is not used as a major therapy. The control interventions will include no therapy, placebo, drug treatment, and other therapies.

#### 2.1.4 Types of outcome measures

##### 2.1.4.1 Primary outcomes

The primary outcomes are the ocular surface disease index (OSDI), the Schirmer test (SIT), the tear meniscus height (TMH), and the tear film break-up time (BUT). The ocular surface disease index questionnaire is used to evaluate the OSDI.^[28,29]^ SIT is used to record measurement of tear secretion by putting the strip in the lower eyelid for 5 minutes.^[30]^ The TMH and BUT (noninvasive first tear break-up time [NIBUTf] and noninvasive average tear breakup time [NIBUTavg]) are measured with a noninvasive ocular surface analyzer.^[31]^

##### 2.1.4.2 Secondary outcomes

The secondary outcome measures will include the following:

1. Quality of life: Evaluated by the Chinese version of Dry Eye Related Quality of Life (CDERQOL) score.^[32,33]^
2. Main symptom scores before and after treatment: Evaluated by the Symptom Assessment in Dry Eye (SANDE) scale.^[34]^
3. Meibomian gland (MG) morphology: MG loss, thickness, and bent angle of the upper and lower lid are measured by non-contact infrared meibography and image analysis.^[35]^
4. Total treatment efficacy rate: Number of patients whose DE improved.

##### 2.1.4.3 Safety outcomes

The safety outcomes will be measured by the incidence and severity of side effects. Any unexpected events that occurred during the studies will be recorded on an adverse event report form.

### 2.2 Search methods for the identification of studies

#### 2.2.1 Electronic searches

Nine medical databases, namely, PubMed, EMBASE, the Cochrane Library, Google Scholar, Web of Science, China National Knowledge Infrastructure (CNKI), China Science and Technology Journal database (VIP), Wanfang Database, and CBM, will be searched from their inception to February 28, 2019, for reviews on moxibustion and DE. The search term will include two parts: that is, moxibustion (eg, direct moxibustion, indirect moxibustion, heat-sensitive moxibustion, moxa burner moxibustion, warm needling, crude drug moxibustion, or natural moxibustion and DE (eg, DE, DE disease, DES, xerophthalmia, keratoconjunctivitis sicca, or keratitis sicca). The equivalent search entries will be used while searching in the Chinese databases. The fully reproducible search strategy provided in Table 1 is for PubMed. This will be appropriately adapted for search in the other databases.

**Table 1:**
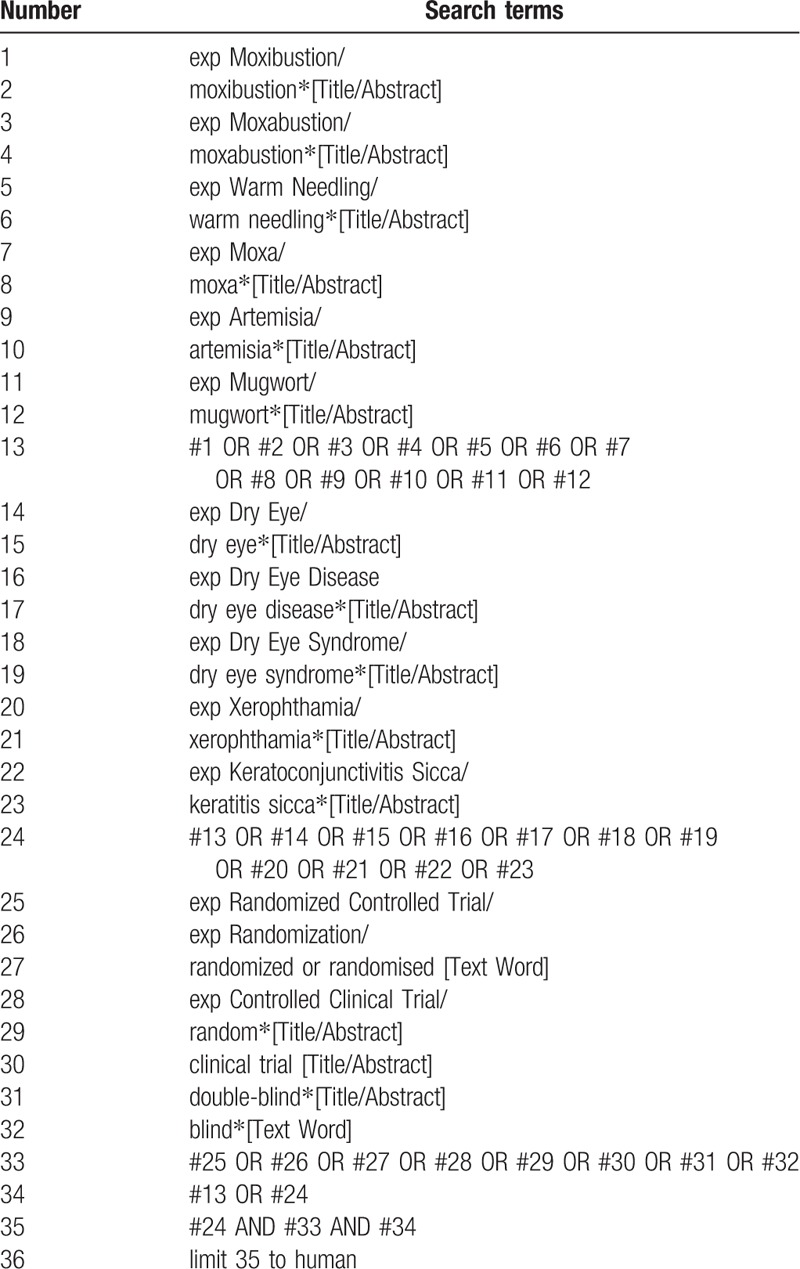
Search strategy used in PubMed database.

#### 2.2.2 Searching other resources

Meanwhile, we will also search PROSPERO, the International Clinical Trials Registry Platform (ICTRP), ClinicalTrials.gov, dissertations, and gray literature to identify systematic reviews or clinical trials related to moxibustion and DE. Relevant journals and conference processes will be manual searched. We will also review papers and bibliographies included in the trials.

### 2.3 Data collection and analysis

#### 2.3.1 Selection of studies

**Figure 1:**
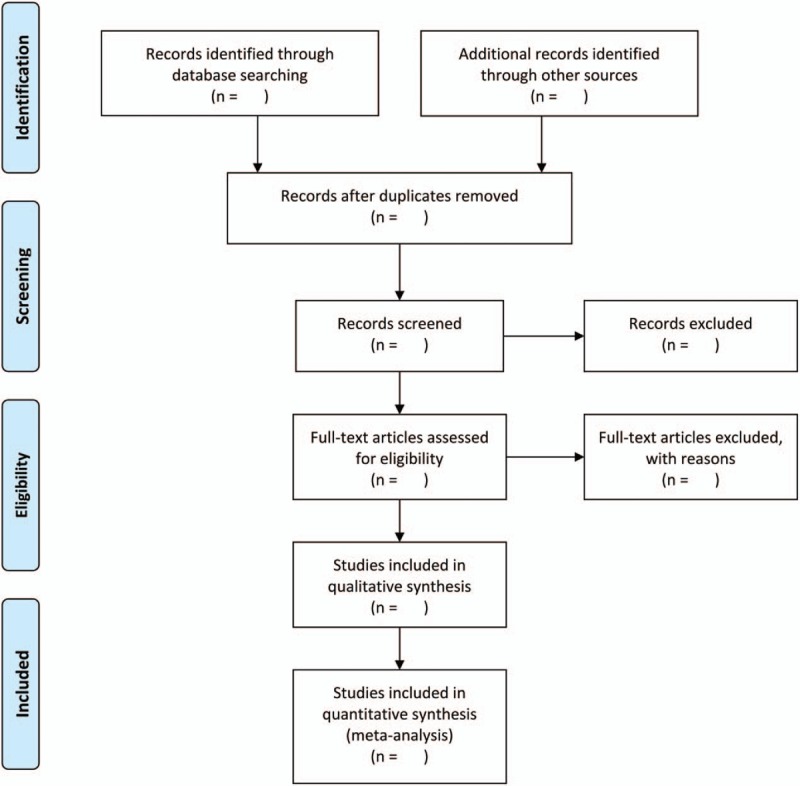
The PRISMA flow chart. PRISMA = Preferred Reporting Items for Systematic Reviews and Meta-Analyses.

We will select studies involved in any form of moxibustion as the sole treatment or as a major therapy. Moxibustion will be classed as the major therapy when the literature suggests that the frequency of application of moxibustion is higher and the time is longer than other intervention methods. Studies only related to human subjects will be included. Two reviewers (Y.X.Z. and M.Q.J.) will independently browse the titles, abstracts, and keywords of all of the retrieved records to distinguish and exclude any obviously irrelevant articles. If these reviewers have disagreements, a third author (M.J.) will make the final decision. The study selection procedure is presented in a PRISMA flow chart (Fig. 1).

#### 2.3.2 Data extraction and management

On the basis of the inclusion criteria, a standard data collection form will be produced before data extraction. EndNote X8 software will be used to manage the records we obtained from electronic databases and other resources. Data will be independently extracted and written on the data collection form by 2 authors (Y.X.Z. and M.Q.J.). If any details of the trial are incomplete, we will contact the appropriate author for more information. The following data will be extracted: study type; title; author; year of publication; journal; country; hospital setting; study design; sample number; dropout number; diagnostic criteria, inclusion and exclusion criteria; age; sex, disease severity, research site; type of moxibustion; acupoints; interventions duration and frequency; outcomes as mentioned above.

#### 2.3.3 Assessment of risk of bias

Two authors (Y.D. and Y.L.Q.) will independently evaluate the risk and bias using the Cochrane risk of bias (ROB) assessment tool.^[36]^ The RevMan software program (V.5.3) will record the selected details of each study.^[37]^

#### 2.3.4 Measures of treatment effect

The risk ratio (RR) and 95% confidence interval (CI) will be used to analyze dichotomous data and measure the treatment effect. A weighted mean difference (WMD) or a standard mean difference (SMD) with 95% CIs will be used to analyze continuous outcomes.

#### 2.3.5 Unit of analysis issue

To avoid a unit of analysis issue, we will only evaluate the first experimental period data of crossover trials. Meanwhile, considering that there are multiple intervention groups in trials, we will combine all analogous groups into a single pair-wise comparison.

#### 2.3.6 Management of missing data

One reviewer (H.M.) will contact the corresponding author or relevant author of an article via e-mail and telephone to obtain any missing data. If there is no reply from the authors, the missing data will be got rid of. In this event, this will be addressed in the “Discussion” section.

#### 2.3.7 Assessment of heterogeneity and data synthesis

Heterogeneity will be tested with a standard chi-square test.^[38]^ A *P* value of less than .10 will indicate that the difference is statistically significant.^[39]^ To quantify the impact of the statistical heterogeneity on the systematic review, the *I*^2^ value will also be calculated. When the heterogeneity tests show little or no statistical heterogeneity, a fixed-effects model will be used. If the *I*^2^ value is more than 50%, a random-effects model will be adopted. We will perform subgroup analysis to explore the potential causes of heterogeneity. We will use V.5.3 for Windows to perform a systematic review and to conduct data synthesis. If meta-analysis is not possible due to lack of clinical studies or heterogeneity, narrative synthesis will be adopted. The Grades of Recommendation, Assessment, Development and Evaluation (GRADE) will be use to assess the meta-analysis findings and describe the strength of evidence.

#### 2.3.8 Assessment of reporting biases

When more than 10 RCTs are selected, the funnel plot and statistic test will be adopted to evaluate reporting biases.

#### 2.3.9 Subgroup analysis

To identify heterogeneity between the included studies, a subgroup analysis will be conducted. The criteria for a subgroup analysis are as follows:

1. Type of control interventions.
2. Type of moxibustion.
3. Species of herb used in the moxibustion treatment.
4. Intervention number, frequency, and duration.

#### 2.3.10 Sensitivity analysis

We will use the ROB tool to assess methodological quality. If low-quality articles are removed, a second meta-analysis will be conducted. The results and effect size of the 2 meta-analyses will be compared and discussed.^[40]^

## 3 Discussion

Dry eye is 1 of the most common ophthalmological diseases, and its incidence rate has increased in recent years. At present, pharmacologic and nonpharmacologic approaches are the main choices for DE.^[5]^ ATs and hormone drugs are the mainstay of DE therapy.^[41]^ However, the transient efficacy of these treatments and their potential adverse effects have limited clinical applications.^[42]^ ATs currently used widely in the market contain preservatives, which can cause damage to corneal epithelial cells. A previous study recruited patients who had treated their DE for at least 6 months with ATs containing preservatives. That study showed that the mean duration of use of ATs containing preservatives was 15.8 ± 12.1 months, and 73.0% of the patients had superficial punctate keratitis (SPK).^[43]^ Topical corticosteroids can produce a number of local adverse side effects, notably cataracts, elevated intraocular pressure (IOP), and increased risk of infection.^[44]^ Thus, nonpharmacological interventions for DE are needed to improve efficacy and reduce side effects. Because of its low side effects and minimal financial burden, moxibustion has been widely used in ophthalmological diseases, such as eyelid disease, optic atrophy, visual fatigue, and refractive error.^[45–47]^

However, the mechanisms of moxibustion in treating DE are still unclear. Despite this, many clinical observations have suggested that moxibustion could promote lacrimal gland secretion and improve tear film stability.^[48–50]^ Therefore, the purpose of this proposed systematic review is to provide a comprehensive review of the effectiveness and safety of moxibustion treatment for DE. We expect that this systematic review will benefit patients with DE, clinicians, healthcare managers, and policy-makers.

## Author contributions

**Conceptualization:** Yingxin Zi, Meiqi Ji, Ming Jin.

**Data curation:** Yingxin Zi, Meiqi Ji, Huan Meng, Ming Jin.

**Formal analysis:** Yingxin Zi, Meiqi Ji.

**Funding acquisition:** Yingxin Zi.

**Investigation:** Ming Jin.

**Methodology:** Yingxin Zi, Meiqi Ji, Yu Deng, Yali Qin, Rui Wang, Huan Meng, Ming Jin.

**Project administration:** Ming Jin.

**Resources:** Yingxin Zi, Meiqi Ji, Huan Meng, Ming Jin.

**Software:** Yingxin Zi, Meiqi Ji, Huan Meng, Ming Jin.

**Supervision:** Yu Deng, Ming Jin.

**Validation:** Yali Qin, Rui Wang, Huan Meng, Ming Jin.

**Visualization:** Yali Qin, Rui Wang, Huan Meng, Ming Jin.

**Writing – Original Draft:** Yingxin Zi, Meiqi Ji, Ming Jin.

**Writing – Review & Editing:** Yingxin Zi, Meiqi Ji, Ming Jin.

Yingxin Zi orcid: 0000-0002-0062-8725.
